# Is the Quality of Judging in Women Artistic Gymnastics Equivalent at Major Competitions of Different Levels?

**DOI:** 10.2478/hukin-2013-0038

**Published:** 2013-07-05

**Authors:** Maja Bučar Pajek, Ivan Čuk, Jernej Pajek, Marjeta Kovač, Bojan Leskošek

**Affiliations:** 1 Faculty of Sport, University of Ljubljana, Gortanova 22, 1000 Ljubljana, Slovenia.; 2 University Medical Centre Ljubljana, Zaloška 2, 1525 Ljubljana, Slovenia.

**Keywords:** aesthetic sports, sport statistics, evaluation, validity, bias

## Abstract

In the present study, the reliability and validity of judging at the European championship in Berlin 2011 were analysed and the results were compared to a different level gymnastic competition – Universiade 2009 in Belgrade. For reliability and consistency assessment, mean absolute judge deviation from final execution score, Cronbach’s alpha coefficient, intra-class correlations (ICC) and Armor’s theta coefficient were calculated. For validity assessment mean deviations of judges’ scores, Kendall’s coefficient of concordance W and ANOVA eta-squared values were used. For Berlin 2011 in general Cronbach’s alpha was above 0.95, minima of item-total correlations were above 0.8, and the ICC of average scores and Armor’s theta were above 0.94. Comparison with Universiade 2009 identified vault and floor scores at both competitions to have inferior reliability indices. At both competitions average deviations of judges from the final E score were close to zero (p=0.84) but Berlin 2011 competition showed a higher number of apparatuses with significant Kendall’s W (5 vs. 2 for Universiade 2009) and higher eta-squared values indicating higher judge panel bias in all-round and apparatus finals. In conclusion, the quality of judging was comparable at examined gymnastics competitions of different levels. Further work must be done to analyse the inferior results at vault and floor apparatuses.

## Introduction

Judging in artistic gymnastics crucially influences sport results. The differences between competitors are often small, especially if the homogenous group such as the world class gymnasts competes at higher level competitions (European and World championships, Olympic Games) ( GymnasticsResultsCom, 2012 ). Here even a small systematic bias of judges may influence final ranks of competitors. Therefore, continuous monitoring of the quality of judging (incorporating reliability and validity) is a necessity.

The present judging Code of Points for women defines 6 judges (or 4 judges for competitions at levels lower than Olympic Games or World Championship e.g. University Games) evaluating exercise execution. This results in the E (execution) score. In addition, 2 judges evaluate exercise content and they provide the D (difficulty) score. E scores range from 10 points down in decrements of 0.1 and D scores go from 0 points rising in increments of 0.1 ( Federation Internationale de Gymnastique - FIG, 2009 ). Since the D score is a joint (consensus) score of both judges who evaluate exercise content, it is impossible to calculate reliability and validity, while for the E score – which is an average score of the middle four (or two) judges – this calculation is possible. It was previously reported that feedback of judging (where judges know other judges’ scores) influences the judges to correct and adjust their scores ( Boen et al., 2008 ). The 2009 Code of Points ( FIG, 2009 ) determines that a judge cannot see other judges’ scores before or after he/she gives his/her own score, but he/she does see the final E score afterwards. The judges therefore produce their score independently, however, some degree of feedback still exists.

Several aspects of judging performance were already described in the past ( Aronson, 1970 ; Bučar Pajek et al., 2011 ; Dallas and Kirialanis, 2010 ; Leskošek et al., 2010 ; Plessner and Schallies, 2005 ; Popović, 2000 ). Ansorge et al. (1978) found bias induced by the position in which female gymnasts appeared in their within-team order; similar results were found by Plessner (1999) . Ansorge and Scheer (1988) found biased judging for judges’ own national team and against immediate competitors’ teams. However, no major attention was devoted to the differences in judging performance between the competitions of different levels. Therefore, the aim of this study was to analyse the reliability and validity of female judging at the European championship in Berlin 2011 and to compare the results with Universiade 2009 in Belgrade. According to the article 5 of 2009 FIG General Judges’ Rules European championship is a level 2 and Universiade a level 3 competition ( FIG, 2009 ). Detailed results of judging performance at Universiade 2009 were already published ( Bučar et al., 2012 ). Here we present the results from Berlin 2011 in detail and compare the crucial results with Universiade 2009.

## Methods

The official book of results was used to obtain E scores. Three sets of analyses were performed; one for each session of the competition. In the first two sets we analysed qualification sessions and all around finals and in the third set we analysed apparatus finals. In each set and on each apparatus 6 judges evaluated E scores.

For each set of analyses, we calculated descriptive statistics for E score, item (individual judge) and scale (all judges together) scores. Distributional statistics (mean and standard deviation) were calculated for individual judge’s E score and mean deviation from final E score of competitors was calculated. This form of deviation is a measure of bias (systematic under-or over-estimation) and can be used to evaluate the validity of judging. Also, mean absolute deviations from E score were calculated for individual judges and used as a measure of reliability.

We calculated the Cronbach’s alpha coefficient for every group of judges on each apparatus and employed this measure to test for consistency of judges as they were evaluating same gymnasts. The corrected item-total correlation (rcorr), i.e. the correlation between individual judge’s scores and total scores, was also calculated.

Other evaluated items were as follows: the Armor’s reliability coefficient, theta (θ), and first and largest eigenvalue (λ1) from the principal component analysis ( Armor, 1974 ). The Armor’s θ is interpreted as a measure of reliability (the proportion of the total variance represented by the between-subject variance). The closer the value is to 1, the lower is the impact of the judges’ errors. Furthermore, two types of intraclass correlation (ICC) were calculated: the single measure and the average measures ICC. ICC coefficients were calculated under one-way random effects model, where judges were conceived as representing a random selection of possible judges, who rate all competitors of interest. ICC equals 1 only when there is no variance due to judges and no residual variance. Additionally, two analyses of between-judges differences were performed: the Kendall’s coefficient of concordance and repeated measures ANOVA. High (statistically significant) values of Kendall’s W indicate systematic bias (under- or overestimation) with at least one of the judges. Through the use of repeated measures ANOVA eta-squared (η2) values were calculated, which represent the proportion of the total variance in dependent variable (scores) explained by the independent variable (judges) and range from 0 to 1. So besides estimating judge bias on an individual level with individual mean deviation from E score (see above), we used eta-squared values to assess the bias for the whole judge panel separately for all sessions and apparatuses.

The study was ethically approved by the European Gymnastics Federation under the supervision of its technical committees. Full blinding of the judges involved was undertaken. To protect the judges’ anonymity we randomly changed their position in the analysis from the book of results. All data were analysed with PASW Statistics v. 18.0.3 software (SPSS Inc., Chicago, IL, USA) whenever possible, otherwise with Microsoft Excel v. 11.0 (Microsoft Corporation, USA).

## Results

The statistics of E scores and number of competitors for all three sessions are shown in Table 1 . Additionally, average D scores were also presented.

The variability of E scores (dispersion) is in general larger for uneven bars and balance beam (except for balance beam in session 3) and is relatively small in the vault. The apparatus finals are the session with the highest scores but not the smallest dispersion. At Berlin’s 2011 competition the difficulty level of performed elements was higher.

In Table 2 , the worst individual deviations in judging for each session and apparatus (all remaining individual judge values were better) were presented. Besides the worst deviations also the smallest values for item-total correlation were indicated as well as the Cronbach’s alpha coefficient for each apparatus.

It can be seen in Table 2 that maximal individual judge mean deviations from the final E score are overall relatively small, all of them below 0.2 score. In terms of measures of common performance for Berlin competition, the all around finals on vault and floor and floor apparatus finals are the apparatuses with the relatively poorest values of Cronbach’s alpha and the smallest values of minimum item-total correlation. However, most of the values are still above 0.8. In all of the parameters shown in Table 1 there were no significant differences between both compared competitions.

**Figure 1:**
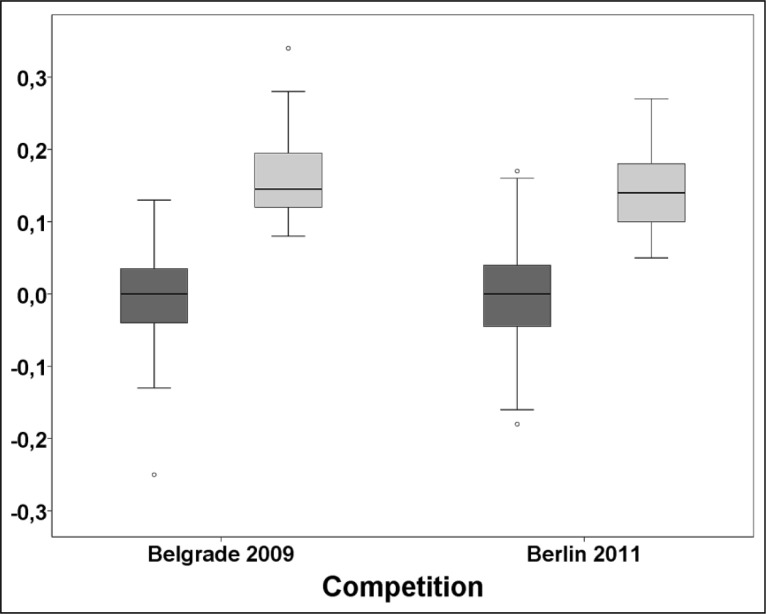
*
Boxplot for mean deviations (a measure of bias, dark grey) and mean absolute deviation (a measure of reliability, light grey) for both compared competitions.
* *
P=0.84 for mean deviations difference between competition and p=0.25 for mean absolute deviation differences between competitions.
*

To compare the bias of judges for Berlin and Belgrade competitions, the differences of mean deviations from E score for both competitions were tested. The boxplots of mean deviations and mean absolute deviations (a measure of reliability) are shown in Figure 1 . No significant differences overall between both competitions were found. When individual sessions were compared, no significant differences were found as well except for mean absolute deviations in apparatuses finals sessions, which showed higher values at Belgrade 2009 competition (median 0.16 vs. 0.13 for Berlin 2011, p=0.006).

**Figure 2:**
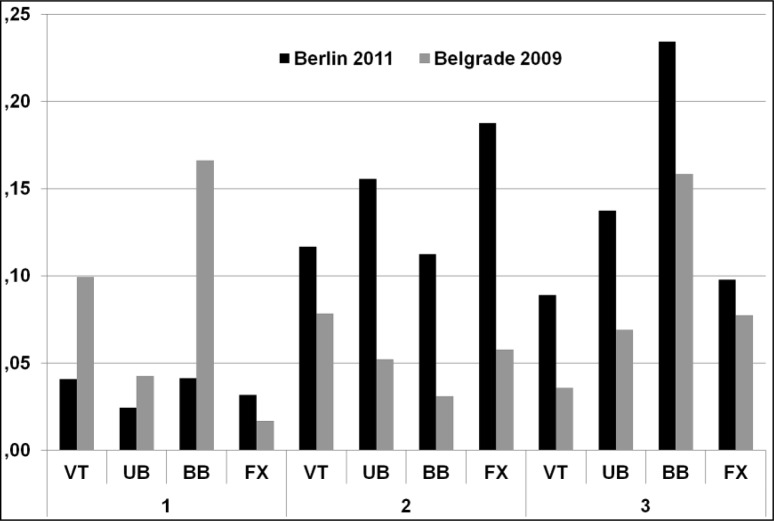
*
The eta-squared values of E-scores for Berlin 2011 and Belgrade 2009 competitions. (1 - qualifications, 2 - all around finals, 3 - apparatus finals;
* *
VT: vault; UB: uneven bars; BB: balance beam; FX: floor)
*

When testing the inter-judge differences with repeated measures ANOVA, eta-squared values representing the bias effect size were calculated – Figure 2 . It can be seen that eta-squared values representing judge panel bias are somewhat higher for Berlin 2011 competition in all-round and apparatus finals sessions.

**Figure 3:**
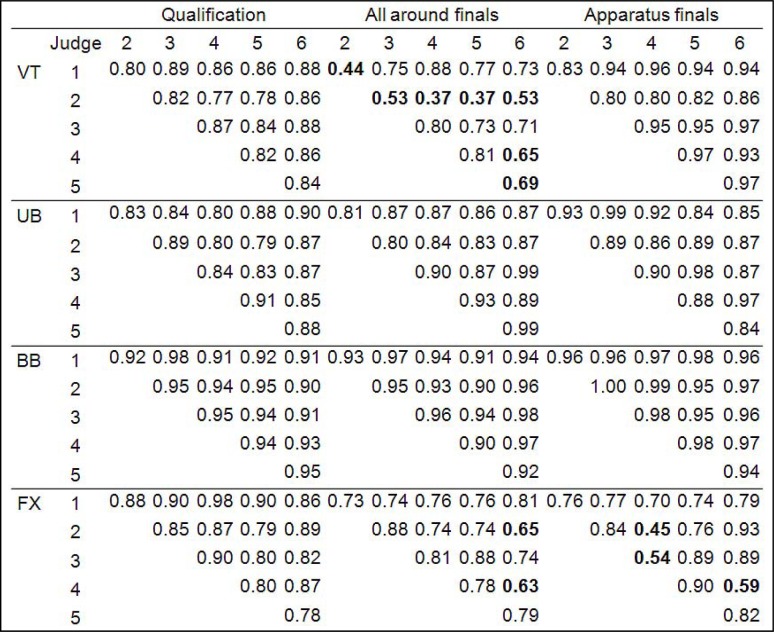
*
Correlation matrix for between-judge correlations.
* *
The remarkably inferior correlations below 0.7 are shown bold.
* *
VT: vault; UB: uneven bars; BB: balance beam; FX: floor.
*

Next, we performed the analysis of between-judge correlations; the Pearson’s correlation coefficients matrix is shown in Figure 3 .

It is evident that most of the correlation coefficients are above 0.8. Again, vault and floor all around finals and floor apparatus final show somewhat inferior correlations. On the basis of this correlation matrix three outstandingly inferior judges (number 2 and 6 in vault all around finals and judge number 4 in floor apparatus finals) can be identified with higher disagreement to others.

Overall measures of inter-judge reliability are shown in Table 3 . For Berlin competition, the relatively poorer concordance of judges on vault and floor all around finals and floor apparatus final can be inferred from the calculated ICC of single values in Table 3 , otherwise the observed ICC values are high - mostly above 0.8. The Armor’s theta coefficient follows quite closely the values of ICC for average values and Cronbach’s alpha coefficient. ICC for single values however shows the highest sensitivity for the deviations in inter-judge agreement and reliability, when compared to other measures (Cronbach’s alpha, ICC for average measures and Armor’s theta). Kendall’s W is statistically significant for the vault and floor in qualification sessions and for all apparatuses except vault in all around finals.

## Discussion

In the present analysis we report the indices of reliability and validity for female judging at one of the highest level competitions – the European championship in Berlin 2011. To the best of our knowledge, this is also the first comparative report of reliability and validity of judging at two major gymnastics events of different levels. Overall, for the European championship the indices of consistency are satisfactory. Except for the vault and floor all around finals and floor apparatus finals Cronbach’s alpha is above 0.95, minima of item-total correlations are above 0.8, and the ICC of average scores and Armor’s theta coefficients are at or above 0.95, which are all good values.

When trying to explain the three inferior reliability results for the above mentioned vault and floor apparatuses it is valuable to inspect the between-judge correlation matrix ( Figure 3 ), as many of the reliability measures of judges’ performance are based on Pearson’s correlations. We can identify three judges whose number of correlation coefficients below 0.7 is three or more (judges 2 and 6 on vault all around finals and judge 4 on floor apparatus final). These judges also show relatively inferior item-total correlation coefficients of 0.5, 0.77 and 0.69, respectively. To further clarify the factors contributing to the observed lower consistency and reliability in vault and floor apparatuses, the comparison to Universiade 2009 is valuable. The lower reliability indices were found for some of the vault and floor apparatuses at that competition as well ( Table 3 ). It seems that vault and floor competitions are outstandingly vulnerable to inferior judging reliability.

We can speculate that the shortage of time available for vault judges to see and mark all the possible deductions is perhaps a source of additional variability in their scores. Average vault takes less than 5 seconds: first flight between 0.06 – 0.17 s, support 0.19 – 0.26 s, second flight up to 1.01, landing up to still standing position 3 s ( Čuk and Karacsony, 2004 ) and judges are expected to mark up to 22 possible items for which the deductions are possible almost in every vault phase. It may well be that some of the deductions are made through the inference with previous experience and this may be the source of additional inter-judge variability. To support this statement, Ste-Marie and Lee (1991) and Ste-Marie et al. (2001) found that memory of pre-processed data has influence on the quality of judging. Furthermore, Ste-Marie (2000) reported that novice judges spent less time watching the gymnast and more time looking at the scoring paper than expert judges and this could be the source of additional variability especially at vault when the time to observe the gymnast is much shorter compared to other apparatuses.

In case of floor exercises additional sources of excess inter-judge variability are in play. For example the judges are expected to make artistry deductions for the lack of creativity of choreography, inability to express idea of the music, inappropriateness of gesture, etc. ( FIG, 2009 ). These are all highly subjective categories and consequently a possible source of additional discordance between judges. Since this explanation is at present no more than speculative it would be valuable in future to analyse specifically the impact of these artistry deductions on the ranking of competitors, especially since the sum of these deductions may reach up to 1.1 points.

Of note, when the overall measures of inter-judge reliability are considered, the ICC for single measures was the measure most sensitive to inter-judge variations. Although current analysis shows that the judging on vault and floor was substandard, this was similar for both Berlin and Belgrade competitions. When all other apparatuses and sessions are considered, it is possible to conclude that good and similar values of reliability indices were found at both competitions and therefore reliability of judging was maintained at both events similarly. This is supported also by similar and non-significantly different values of mean absolute deviations ( Figure 1 ) which are a measure of reliability on individual judge level. This is in accordance with the comparisons of reliability reported in different judging analyses at single competitions over time ( Bučar et al., 2011 ; Leskošek et al., 2010 ).

When examining validity, the ideal test of validity would have to implement a comparison of concrete judging with the gold standard of judging performance; however no such gold standard currently exists. It is possible however, to focus on a special case of validity, which deals with the presence of systematic over or under rating or scoring of competitors - what is also called bias ( Bučar et al., 2011 ). With bias we refer to repetitive under- or over-estimation of particular judges. To examine this bias, we have used mean deviations from E score, Kendall’s coefficient of concordance and repeated measures ANOVA eta-squared values. It was somewhat surprisingly to find consistently higher values of eta-squared values throughout all-round finals and apparatus finals at Berlin 2011 competition. Berlin 2011 was a competition of a relatively higher level and hosted judges in average of senior ranking. Perhaps this result implicates that senior judges are more adherent to their own criteria and were adjusting their scores to the mean of the group in a smaller extent. The process of adjusting towards the mean of the group was found to be operative in gymnastic judges ( Boen et al., 2008 ), however no data exist on the comparison of the magnitude of this process for judges of different levels.

In conclusion, present analysis showed in general acceptable judging reliability at two different levels of female gymnastic competitions. The comparison of reliability indices brought attention to vault and floor apparatuses, which seem more vulnerable to deviations from high reliability indices found in other apparatuses. Although we can provide some explanatory factors for this, further work is needed to firmly establish the causes and to find ways for improvement. When bias was analysed, we found equivalent values of mean deviations from final E scores for both competitions. However, we found more cases of significant Kendall’s concordance coefficients and higher eta-squared values at Berlin 2011 contest, which is a relatively higher level competition. It can be concluded that the quality of judging in general was well maintained at examined gymnastics competitions of different levels, but in future there must be further work done to analyse the inferior results at vault and floor apparatuses and test the solutions for improvement.

## Figures and Tables

**Table 1:** *
Statistics of E scores with mean and standard deviation (SD), comparison to Universiade 2009 results
*

Session	apparatus	N	N *	Mean	SD	Mean *	SD *	D score	D score *
1	VT	97	71	8.40	0.33	8.35	0.42	5±0.5	4.8±0.48 ^†^
	UB	75	46	7.40	0.80	7.28	1.22	4,97±1,16	4,86±1
	BB	79	47	7.25	0.97	7.28	1.02	5.2±0.71	4.81±0.7 ^†^
	FX	73	47	7.69	0.59	7.92	0.49	5±0.6	4.83±0.56

2	VT	24	24	8.55	0.20	8.38	0.49	5.3±0.52	4.95±0.37 ^†^
	UB	23	24	7.78	0.53	7.19	1.30	5.57±0.41	4.83±1 ^†^
	BB	23	24	7.86	0.80	7.28	1.05	5.6±0.46	4.96±0.71 ^†^
	FX	23	24	8.11	0.39	7.68	0.57	5.3±0.3	4.98±0.44 ^†^

3	VT	16	16	8.57	0.43	8.75	0.14	5.6±0.54	5.19±0.51
	UB	8	8	8.18	0.56	8.40	0.63	6.13±0.33	6.06±0.64
	BB	8	8	7.96	0.26	8.23	0.65	5.9±0.34	5.61±0.5
	FX	8	8	8.44	0.58	8.48	0.20	5.7±0.24	5.43±0.3

* *Results are from Universiade 2009 in Belgrade ( Bučar et al, 2012 ).*

*Session 1,2,3: qualifications, all around finals, apparatus finals; VT: vault;*

*UB: uneven bars; BB: balance beam;*

*FX: floor; N: number of competitors.*

† *the difference between D scores in both competitions is significant with p<0.05*

**Table 2:** *
The performance of individual judges
*

Session	App	Dev max	Dev max *	Ab dev max	Ab dev max *	R min	R min *	Cα	Cα *
1	VT	−0.05	−0.09	0.13	0.15	0.86	0.83	0.97	0.94
	UB	0.08	0.09	0.27	0.28	0.88	0.92	0.97	0.98
	BB	0.11	−0.25	0.25	0.34	0.94	0.92	0.99	0.97
	FX	−0.05	−0.03	0.20	0.15	0.88	0.88	0.97	0.95

2	VT	−0.12	−0.07	0.24	0.11	0.50	0.93	0.90	0.98
	UB	−0.18	0.07	0.26	0.20	0.87	0.97	0.97	0.99
	BB	−0.1	−0.08	0.18	0.24	0.93	0.92	0.99	0.98
	FX	0.16	0.06	0.21	0.14	0.80	0.91	0.94	0.97

3	VT	−0.07	−0.04	0.14	0.16	0.84	0.2	0.98	0.71
	UB	0.13	−0.08	0.18	0.20	0.91	0.9	0.98	0.98
	BB	0.17	−0.13	0.21	0.22	0.97	0.89	0.99	0.98
	FX	−0.08	0.12	0.14	0.21	0.69	0.36	0.94	0.83

* *Presented results are from Universiade 2009 in Belgrade ( Bučar et al., 2012 ).*

*There were no statistically significant differences when values from Berlin and Belgrade competition were tested with the Mann-Whitney’s test.*

*Session 1,2,3: qualifications, all around finals, apparatus finals;*

*VT: vault; UB: uneven bars; BB: balance beam; FX: floor;*

*Dev max: maximal judge average deviation from E score, Ab dev max: maximum of average absolute deviation from E score;*

*R min: minimum of corrected item-total correlation of individual judges;*

*Cα: Cronbach’s alpha coefficient.*

**Table 3:** *
Overall measures of inter-judge reliability
*

Session	Apparatus	ICC single	ICC single *	ICC average	λ_ 1 _	Armor’s theta	Kendall’s W	p(W)	Kendall’s W *	p(W) *
1	VT	0.83	** 0.79 **	0.97	5.21	0.97	** 0.04 **	** <0.01 **	** 0.10 **	** <0.01 **
	UB	0.84	0.91	0.97	5.23	0.97	0.01	0.72	0.04	0.16
	BB	0.92	0.88	0.99	5.63	0.99	0.03	0.06	** 0.07 **	** 0.02 **
	FX	0.84	0.83	0.97	5.23	0.97	** 0.05 **	** <0.01 **	0.01	0.65

2	VT	** 0.57 **	0.91	0.89	4.29	0.92	0.06	0.19	0.08	0.11
	UB	0.84	0.97	0.97	5.35	0.98	** 0.11 **	** 0.03 **	0.07	0.17
	BB	0.93	0.92	0.99	5.70	0.99	** 0.11 **	** 0.03 **	0.01	0.78
	FX	** 0.70 **	0.89	0.93	4.80	0.95	** 0.17 **	** <0.01 **	0.04	0.43

3	VT	0.90	** 0.30 **	0.98	5.56	0.98	0.08	0.28	0.05	0.59
	UB	0.87	0.88	0.98	5.43	0.98	0.15	0.32	0.06	0.81
	BB	0.95	0.87	0.99	5.84	0.99	0.24	0.09	0.12	0.43
	FX	** 0.75 **	** 0.47 **	0.95	4.76	0.95	0.10	0.55	0.10	0.57

* *Presented results are from Universiade 2009 in Belgrade ( Bučar et al., 2012 ).*

*For ICC single the correlation coefficients below 0.8 are put in bold.*

*For Kendall’s coefficient of concordance the significant values (expressing bias in the judge panel) are put in bold.*

*Session 1,2,3: qualifications, all around finals, apparatus finals;*

*VT: vault; UB: uneven bars; BB: balance beam; FX: floor;*

*ICC single (average): intra-class correlation for single (average) scores;*

*λ _1_ : first eigenvalue from the principal component analysis;*

*p(W): p value of Kendall’s W.*
